# Coronary vessel wall assessment after Kawasaki Disease

**DOI:** 10.1186/1532-429X-13-S1-P235

**Published:** 2011-02-02

**Authors:** Tarique Hussain, Sarah Peel, Aphrodite Tzifa, Thomas Krasemann, Gerald Greil, Rene Botnar

**Affiliations:** 1 King's College London, London, UK

## Introduction

2D cross-sectional Dual-Inversion-Recovery (DIR) black-blood imaging of the coronary arteries has been used to assess Kawasaki Disease (KD) (Greil et al. 2007). A 3D coronary black-blood approach, however, has been shown to provide better coverage of the coronary tree (Botnar et al 2001). Furthermore, uptake of gadolinium contrast agent in coronary walls may indicate on-going inflammation or vessel wall fibrosis (Maintz et al, 2006).

## Aim

To investigate whether in-plane 3D coronary vessel wall imaging utilizing a local-inversion technique and spiral image acquisition, in combination with contrast-enhanced inversion-recovery (IR) imaging allows more comprehensive assessment of KD.

## Methods

Patients with previous coronary aneurysms due to KD undergoing routine CMR evaluation had additional vessel wall imaging before and after contrast administration (0.2 mmol/kg gadopentetate dimeglumine). All examinations were performed on a 1.5T MR-system and the coil was selected according to patient size (two-element/five-element). Coronary MRA was followed by a targeted, free-breathing, ECG-triggered 3D vessel wall sequence using local inversion and spiral image acquisition (spatial resolution=0.76x0.76x2mm; TE/TR=2/29ms, FA=90°). Areas of vessel wall thickening on in-plane imaging were then further evaluated with through plane 2D DIR vessel wall technique.

Post-contrast imaging was performed using a free-breathing, ECG-triggered, 3D IR segmented gradient-echo (TFE) sequence (spatial resolution=1.25x1.25x3mm, TE/TR=1.4/3.5ms, FA=30° & TI chosen to null blood using Look-Locker).

## Results

**Figure 1:**
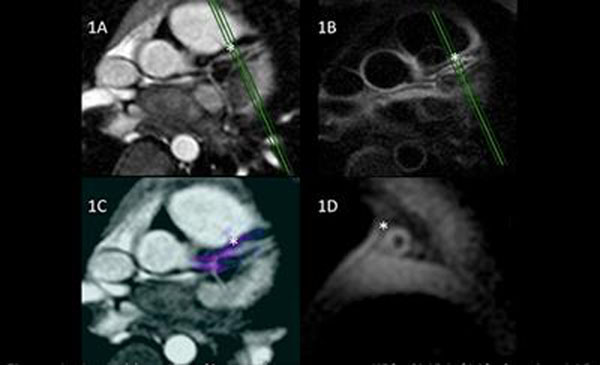
4yrs old patient (9 months post-acute KD). CMRA (1A) showing LAD aneurysm (*). 3D in-plane local inversion black blood of LAD (1B). Delayed Enhancement overlay (purple) on CMRA showing enhancement of aneurysm (1C). Green marker on 1A & 1B shows position of DIR cross-section (1D).

**Figure 2:**
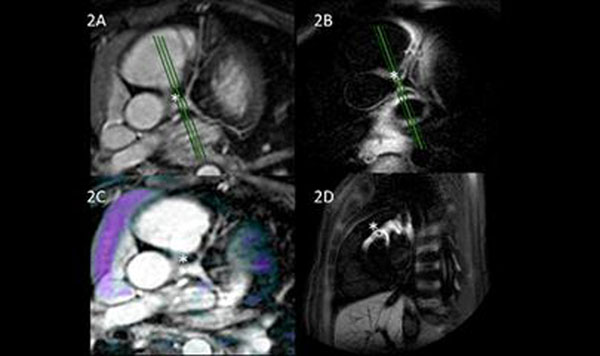
4yrs old patient (3 yrs post-acute KD). CMRA (2A) showing Left Main extasia (*). 3D in-plane local inversion black blood of left main (2B). Delayed Enhancement overlay (purple) on CMRA showing no persisting enhancement (2C). Green marker on 2A & 2B shows position of DIR cross-section (2D).

13 CMR examinations were performed in 12 children (7 male, age=2-19yrs; HR=60-110bpm; 6 weeks-18 years post-acute KD). 6 cases showed persistent aneurysms; 4 cases had coronary ectasia and the remaining resolved aneurysms had a normal lumen. One case showed a mild LAD stenosis shown on CMRA and confirmed on conventional angiography. Complete black blood coverage of the affected portions of the coronary arteries was achieved using the new 3D in-plane local-inversion technique. Targeted 2D cross-sectional imaging showed that aneurismal segments had greater wall thickness (mean 0.87mm) than ectatic segments (0.42mm) or resolved aneurysms (0.31mm). Delayed enhancement of the coronary vessel wall was only present in three cases (all of whom had recent KD <2yrs ago). Figures 1 and 2.

## Conclusion

Our data suggests that inflammation of the coronary vessel wall in Kawasaki disease may persist for up to 2 years post-acute insult. Vessel wall remodeling seems to have occurred effectively in the resolved aneurysms studied here. 3D in-plane local-inversion black-blood imaging gives more complete coverage of affected segments and allows targeting of cross-sectional imaging of thickened areas. The protocol outlined here gives a more comprehensive assessment of affected coronaries after KD.

